# Effect of 2.5D haptic feedback on virtual object perception via a stylus

**DOI:** 10.1038/s41598-021-98589-2

**Published:** 2021-09-23

**Authors:** Gyuwon Kim, Donghyun Hwang, Jaeyoung Park

**Affiliations:** 1 Korea Institute of Science and Technology (KIST), Center for Intelligent and Interactive Robotics, Seoul, 02792 Korea; 2 Department of Mechanical Engineering, Korea University, Seoul, 02841 Korea; 3 Department of Computer Engineering, Hongik University, Seoul, 04066 Korea

**Keywords:** Psychology, Engineering

## Abstract

As touch screen technologies advanced, a digital stylus has become one of the essential accessories for a smart device. However, most of the digital styluses so far provide limited tactile feedback to a user. Therefore we focused on the limitation and noted the potential that a digital stylus may offer the sensation of realistic interaction with virtual environments on a touch screen using a 2.5D haptic system. Thus, we developed a haptic stylus with SMA (Shape Memory Alloy) and a 2.5D haptic rendering algorithm to provide lateral skin-stretch feedback to mimic the interaction force between fingertip and a stylus probing over a bumpy surface. We conducted two psychophysical experiments to evaluate the effect of 2.5D haptic feedback on the perception of virtual object geometry. Experiment 1 investigated the human perception of virtual bump size felt via the proposed lateral skin-stretch stylus and a vibrotactile stylus as reference. Experiment 2 tested the participants’ ability to count the number of virtual bumps rendered via the two types of haptic styluses. The results of Experiment 1 indicate that the participants felt the size of virtual bumps rendered with lateral skin-stretch stylus significantly sensitively than the vibrotactile stylus. Similarly, the participants counted the number of virtual bumps rendered with the lateral skin-stretch stylus significantly better than with the vibrotactile stylus. A common result of the two experiments is a significantly longer mean trial time for the skin-stretch stylus than the vibrotactile stylus.

## Introduction

Since the introduction of the Linus Write-Top computer in the 1980s, a digital stylus has been one of a smart device’s most essential accessories. The digital stylus provides a user experience similar to writing with a real pen, serving a natural and efficient input functionality. As the touch screen technologies advanced, the digital stylus can provide a more realistic writing experience than ever with a reduced time lag. However, most digital styluses so far offer either no or limited tactile feedback so that a user would feel an unrealistic slip between the pen tip and the tablet screen. When a user strokes over a real object’s bumpy surface, the skin at the fingertip will be stretched in the lateral direction, stimulating SA2 type mechanoreceptors^1^. Then, a realistic haptic rendering of stroking sensation via a stylus will first require a 2.5D haptic rendering algorithm, which converts a virtual object’s geometry on a flat surface to 3D tactile information. Also, a lateral haptic feedback for a stylus is necessary to emulate the skin-stretch at the fingertip as a user strokes over a bumpy surface.

Previous studies indicate that the lateral skin stretch feedback can affect human perception of an object’s global properties, including weight and stiffness. For example, the lateral cutaneous feedback to the fingertip in the gravity direction is found to create the sensation of a virtual object’s weight^2,3^. Also, when both force and cutaneous feedback is presented to a user’s fingertip holding a virtual object, s/he can feel an additive weight due to the cutaneous feedback. Moreover, a recent study found that the lateral cutaneous feedback in the direction of object motion can create additive weight sensation^4^. Finally, Quek and Okamura applied the lateral feedback to a stylus and demonstrated that the feedback could modulate the perceived stiffness of a virtual surface^5,6^. However, it is hard to find studies investigating the effect of lateral feedback for a stylus to feel the sensation of a virtual object’s surface bumpiness rendered on a flat surface.

Multiple studies proposed 2.5D haptic rendering methods and witnessed their effectiveness in providing 3D objects’ geometric information via a flat medium. A popular form of the 2.5D haptic feedback display is a pin-array to represent the surface shape of a virtual object. A pin-array type 2.5D haptic display modifies pins’ height to be matched to the virtual object surface swept by a hand. For example, Follmer et al. proposed a tabletop display that can change its surface shape on which a user can achieve 2.5D geometry information of target object with bare hands^7^. They demonstrated that the proposed haptic display could benefit the interaction with the virtual environment and the 3D modeling workflow of those with blindness and visual impairment^8,9^. In the meanwhile, some researchers proposed methods to represent tactile information on a flat surface. Kim et al. proposed a haptic system that could provide 2.5D geometric information with vibrotactile feedback to a user’s fingertip^10^. Sato et al. used a tendon-driven mechanism to provide a user with lateral tactile feedback on a flat surface^11,12^. However, such methods were limited in imparting an object’s geometric information on a flat surface with lateral haptic feedback.

**Figure 1:**
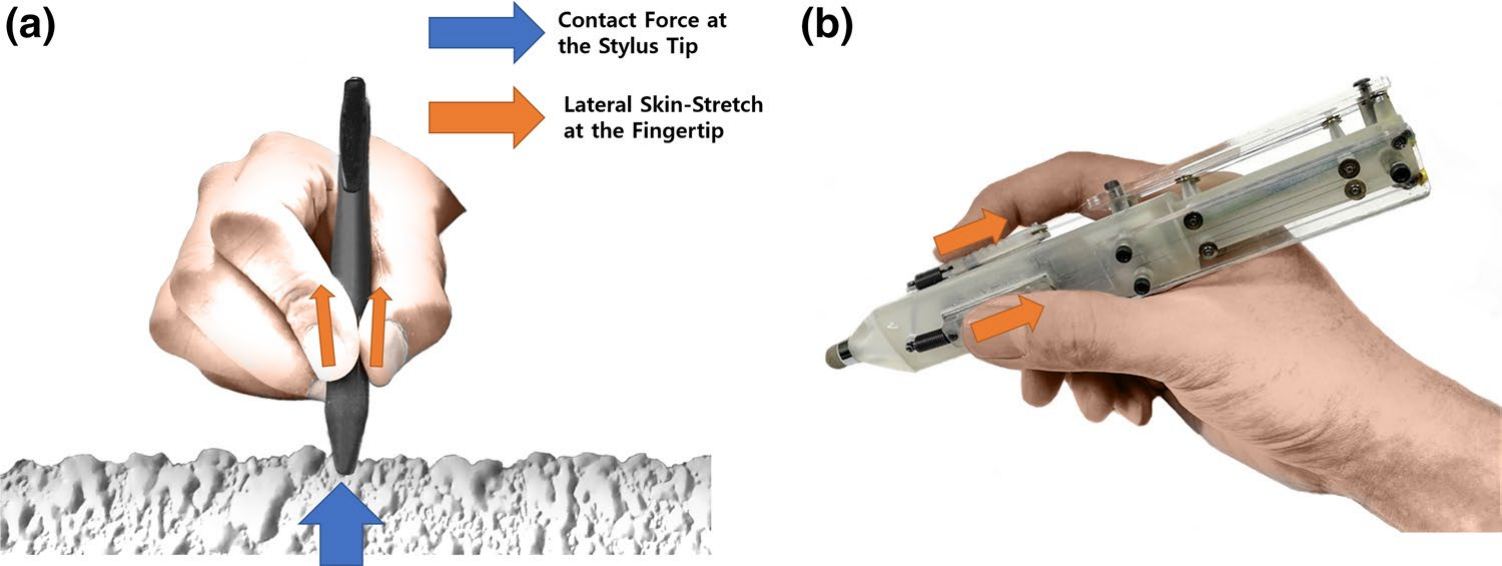
Description of lateral skin-stretch and the experimental apparatus to render the skin-stretch feedback. (**a**) When a stylus strokes over a bumpy surface, the contact force at the tip creates the lateral skin-stretch at the fingertips. (**b**) A haptic stylus to generate the lateral skin-stretch feedback with SMA actuators.

This paper investigates the effect of 2.5D haptic feedback on the perception of a virtual object’s geometry felt via a stylus. We selected the lateral skin-stretch feedback considering its advantage over another form of haptic feedback for a stylus. The pin-array can stimulate the RA and SA1 type mechanoreceptors to provide detailed distributed tactile information^13,14^. However, such a haptic display increases the actuator thickness, which drastically hinders the useability of the stylus. The vibrotactile actuators have advantages such as compact size and low power consumption. However, it is supposed to stimulate only the FA2 mechanoreceptors^1^. Previous studies on lateral haptic feedback mainly focused on its effect on the perception of virtual object property felt with bare fingers. On the other hand, the present study focuses on lateral feedback on the perception of virtual object geometry felt via a stylus stroking over a flat surface. As a reference stimulus, we chose vibrotactile feedback since it is the most widely used haptic feedback for daily applications, including smartphones and tablets. When a user strokes over a bumpy surface with a pen, the contact force will be transferred to the fingertips in the lateral direction (Fig. 1a). Thus, we hypothesize that the lateral skin-stretch feedback will be more effective for human perception of a virtual bump for 2.5D rendering than the reference stimulus, vibrotactile feedback. Also, different types of haptic feedback may have a different effect on the task completion time. We tested our hypothesis with experiments that evaluated human subjects’ ability to perceive virtual bumps rendered by lateral skin-stretch feedback and vibrotactile feedback via a stylus over a flat surface. We designed and built an SMA (Shape Memory Alloy) driven haptic stylus to minimize the weight and the noise while maximizing the lateral force for the skin-stretch feedback (Fig. 1b). For both experiments, a participant stroked over a screen with two different types of haptic styluses that could render either lateral skin-stretch or vibrotactile feedback. An identical haptic rendering algorithm was used for the two experimental conditions. Experiment 1 measured the discrimination threshold for the perceived size of virtual bumps rendered by the two different haptic feedback. Experiment 2 tested the participant’s ability to count the number of virtual bumps that s/he felt as s/he stroked over a screen.

Therefore, the goals of this study are (1) to propose a *2.5D haptic feedback stylus providing lateral skin-stretch feedback to a user’s fingertip*, and (2) to evaluate *the effect of the 2.5D haptic feedback on the perception of a virtual object’s geometric features* and *its efficiency in terms of task completion time*. The following section describes the experimental setup for virtual object size perception and the object counting task.

## Methods

We conducted two human perception experiments to evaluate the effect of 2.5D haptic feedback on virtual object perception. First, this section describes the experimental system overview, the experimental apparatus, and the haptic rendering algorithm. Then, we illustrate the experimental paradigms for the two experiments.

We received approval regarding the experimental methods from the KIST (Korea Institute of Science and Technology) Institutional Review Board (IRB approval No.2020-020) which were carried out by following the Declaration of Helsinki for research involving human subjects. We obtained informed written consent from all the participants.

### Experimental setup

**Figure 2:**
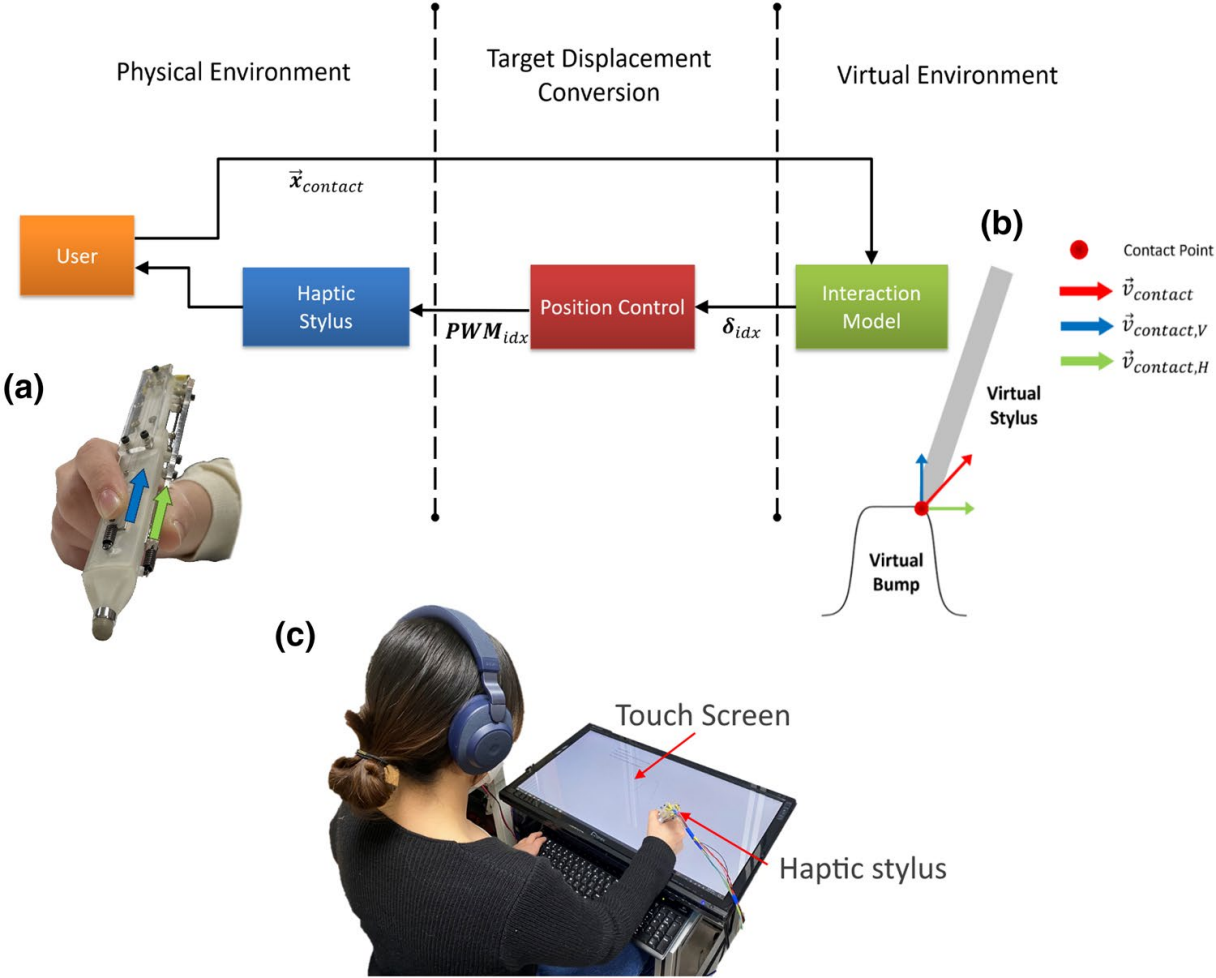
The haptic system architecture for the experiments. When a user strokes a haptic stylus over a touch screen, the contact force at a virtual stylus tip is calculated with a collision detection algorithm (Virtual Environment). Then, the amount of haptic feedback is converted from the contact force (Target Displacement Conversion). Then, a user can feel the tactile stimuli from the haptic stylus. (**a**) A haptic stylus can provide lateral skin-stretch feedback to a user’s fingers. (**b**) The virtual interaction model calculates the vertical and horizontal contact forces. (**c**) The experimental setup consisting of the touch screen and the haptic stylus.

We set up a haptic system where a user can feel virtual bumps via a virtual stylus as s/he strokes over a touch screen for the experiments. Figure 2 shows the overall experimental system architecture, including the virtual environment, skin-stretch displacement conversion block, and the physical environment. We built two types of haptic styluses to test our hypothesis, an SMA-driven stylus for lateral skin-stretch feedback and a vibrotactile haptic stylus. The two styluses were designed to have similar weights to conduct experiments under the same conditions, and they could provide tactile feedback to the user’s thumb, index, and middle fingers. When a participant strokes a haptic stylus over a touch screen, the tip position is mapped to that of a virtual stylus (Fig. 2c). As the contact position changes, a haptic rendering algorithm simultaneously calculates the contact force $\varvec{v}_{contact}$ between the virtual stylus tip and a virtual bump, which is divided into vertical and horizontal components, $\varvec{v}_{contact,V}$ and $\varvec{v}_{contact,H}$. The contact force vectors are then mapped to the magnitude of haptic stimuli depending on the type of the haptic stylus (Fig. 2b). Finally, the haptic stylus is actuated to provide the user with the desired magnitude of haptic feedback to the fingers (Fig. 2a).

**Figure 3:**
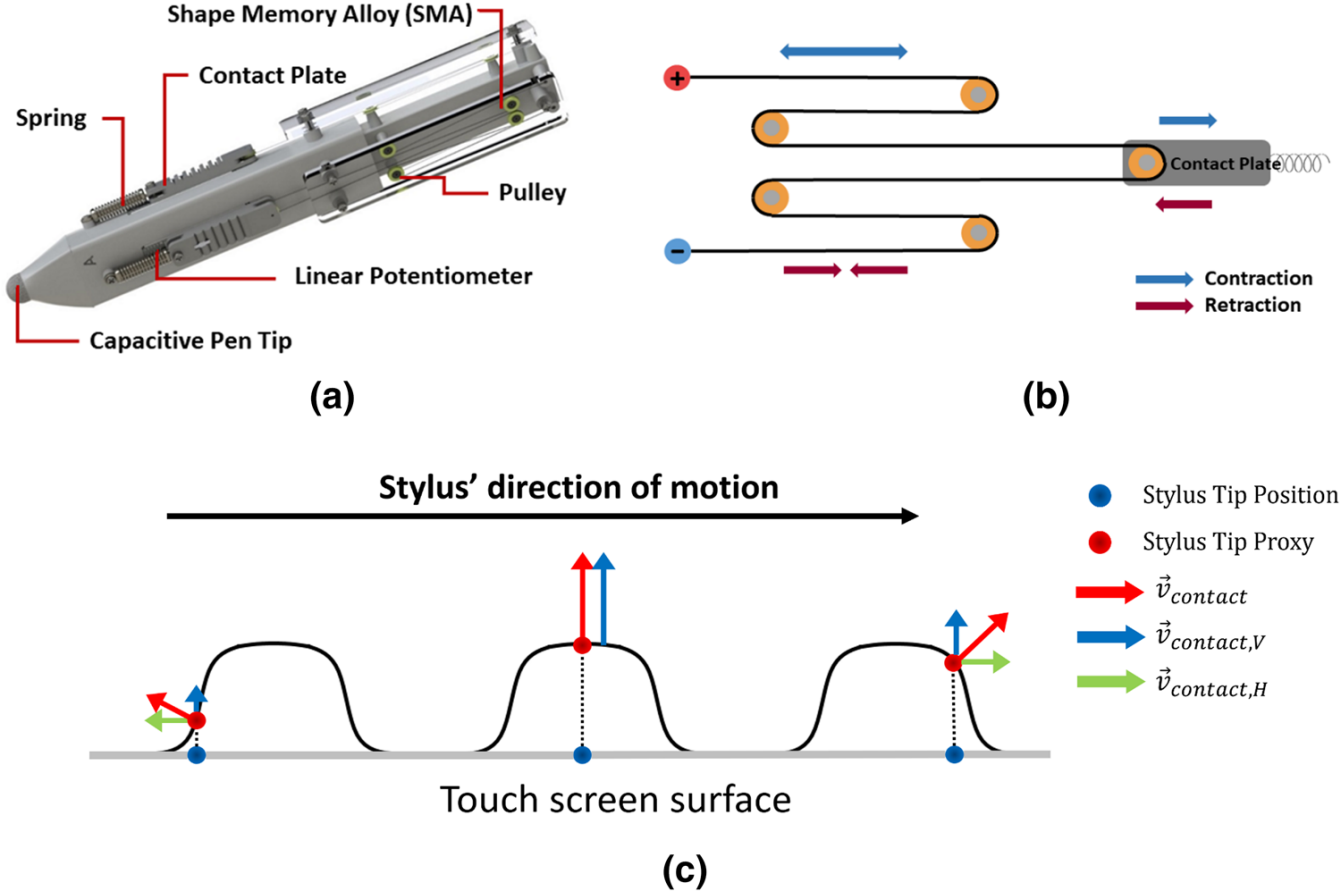
The haptic interface design and haptic rendering principle. (**a**) A CAD design and components of the SMA haptic stylus. (**b**) The working principle of the SMA tendon wound on an actuator unit. Red and blue arrows show the directions of the tendon retraction and contraction, respectively. (**c**) Haptic rendering concept for the contact force between the stylus tip and a virtual bump. The blue and red circles mean the stylus tip position on the touchscreen and its virtual proxy position mapped on the virtual bump. The red, blue, and green arrows indicate the contact normal vector, vertical, and horizontal vector components.

We designed two types of haptic styluses by the type of stimuli, SMA-driven lateral skin-stretch feedback and vibrotactile feedback. Figure 3a shows the SMA stylus to provide lateral cutaneous feedback to the three fingers—thumb, index, and middle fingers—which humans generally use while using a pen. The device is in the shape of a long triangular column where a contact plate is placed on each side of the stylus to provide lateral skin-stretch feedback. We designed the stylus as thin and light as possible while keeping the required mechanism for haptic feedback possible. (For more detailed specifications of the pen, see Supplementary Table 1.) A conductive pen tip is installed at the end of the stylus to be sensed by a touch screen. The actuator unit consists of an SMA tendon/housing part, a spring for restoring, and a contact plate part (Fig. 3b). We used a 0.1 mm thin Flexinol (Dynalloy Inc., Canada) which is a tendon shaped SMA made out of nickel-titanium. The SMA wire thinner than 0.1 mm tends to be fragile and thicker tends to consume higher power. Also, the reaction time under 0.1 A shows less than 0.2 s^15^ (Supplementary Fig. 1b). The tendon is wound around five pulleys to ensure smooth contraction and retraction movement while its endings are fixed to two electrodes. An acrylic cover is placed over the tendon to avoid a user touching the SMA mechanism directly. The contact plate part comprises a bumpy contact plate and a linear potentiometer, which has the total travel distance of 20 mm. The SMA was controlled with proper Pulse Duty Cycle (PDC) (Supplementary Fig. 1e,f), and the power consumption was significantly lower than that of a small servo motor widely used in the conventional multi-axial pen type devices. When heated with a DC voltage application, the tendon contracts, and thus the contact plate moves to the direction opposite to the stylus tip (red arrow in Fig. 3b). When the voltage is lowered, the SMA tendon is cooled down so that the contact plate moves toward the stylus tip with the spring restoring force (blue arrow in Fig. 3b). The vibrotactile stylus has the same dimension as the SMA stylus. It has a contact plate on each side, installed with a dynamic vibration motor (DVM1034, Motorbank Co., Korea) with a vibrating frequency of 233 Hz to provide vibrotactile feedback to a fingertip. A conventional response time of the vibration motor is 50 ms^16^. A haptic motor driver (DRV-2605, Texas Instruments Inc., U.S.A.) controlled the intensity of the vibration for each actuator.

Figure 3c shows the haptic rendering scheme to calculate the magnitude of tactile feedback to the fingertip. We constructed virtual bump contours with the 3rd degree non-uniform rational B-Splines (NURBS) to create a smooth surface^17^. As a stylus moves over a touch screen, its tip position on the touch screen (blue dot in Fig. 3c) is mapped to a position on the surface of the virtual bump, the virtual proxy^18^ (red dot in Fig. 3c). Given the virtual proxy position, the contact normal vector $\varvec{v}_{contact}$ can be readily calculated from the 1st derivative function of the NURBS surface. Next, its magnitude is updated from the height of the proxy from the touch screen surface, i.e., the distance between the proxy and the stylus tip position. Then, the contact normal vector can be divided into a vertical and horizontal components, $\varvec{v}_{contact,V}$ and $\varvec{v}_{contact,H}$ in terms of the stylus velocity on the workspace, $\varvec{v}_{stylus}$. The haptic stimulus intensity, $I_{middle}$, $I_{thumb}$, and $I_{index}$ are then decided from the contact vector’s vertical and horizontal components as follows,

1 $$\begin{aligned} \left\{ \begin{array}{cl}I_{middle}&{}=I_{max}\frac{\left| \varvec{v}_{contact,V}\right| }{\left| \varvec{v}_{contact,V,max}\right| }\\ \underset{idx \in \left\{ thumb, middle\right\} }{I_{idx}}&{}=I_{max}\frac{\left| \varvec{v}_{contact,H}\right| }{\left| \varvec{v}_{contact,H,max}\right| } \end{array} \right. \ \end{aligned}$$

where $I_{max}$, $\varvec{v}_{contact,V,max}$, and $\varvec{v}_{contact,H,max}$ indicate the maximum intensity of the haptic stimulus, the maximum values of the contact normal vector’s vertical and horizontal components, respectively. The values for $I_{max}$ were 20 mm for the skin-stretch stylus and 1.75 g for the vibrotactile stylus, which are over the absolute threshold at the fingertip^19–21^. We set $\varvec{v}_{contact,H,max}$ as 4 cm, which means that a virtual bump with the height of 4 cm can generate a haptic stimulus with the intensity $I_{max}$.

### Experiment 1: Haptic perception of 2.5D virtual bump size rendered by a haptic stylus

The goal of Experiment 1 is to evaluate human ability in bump size discrimination for the two types of 2.5D haptic styluses. The experiment was designed to derive Weber fraction for the two experimental conditions as a measure for bump size perception. A standard one-interval two-alternative-forced-choice (1I-2AFC) or a yes-no experimental paradigm was adopted, which estimates the just noticeable difference (JND)^22^. From the signal detection theory (SDT), the JND is calculated from the sensitivity index $d'$, which indicates the perceptual distance between reference stimulus $\alpha _0$ and comparison stimulus $\alpha _0+\Delta \alpha$. We obtained $d'$ from response matrix, the difference between the hit rate (*H*) and the false alarm rate (*F*) , where $z\left( \cdot \right)$ denotes z score function:

2 $$\begin{aligned} d'=z\left( H\right) -z\left( F\right) . \end{aligned}$$

The JND value can be defined as $(\Delta \alpha )_0$ increment when $d'$ equals to 1. Then, by assuming the linearity between $d'$ and $\Delta \alpha$, we could derive Weber fraction from following equation:

3 $$\begin{aligned} \sigma _s=\left( \Delta \alpha \right) _0/\alpha _0, \end{aligned}$$

Before initiating the main phase, a training session was given to a participant, allowing him/her to get familiarized with the experimental stimuli, reference, and comparison stimuli. In the training session, the participant could see and feel each size of bumps. As the participant drew a straight line on the screen, haptic feedback of the 2.5D feature was rendered to her/his fingers with a haptic stylus. Also, a corresponding figure of a given experimental bump was shown on the screen for better comprehension. There was no limit in time and number of examinations in the training session so that the participant could sufficiently feel the stimulus. During the main experiment, virtual bumps were not visually displayed, but there was only haptic feedback available. One of the two experimental stimuli was presented on each trial at a random location with an equal a a priori probability of 0.5 (2 alternatives). For the given stimuli, the participant was asked to type “0” for the reference stimulus and “1” for the comparison stimulus, indicating what s/he actually felt. Then, by pressing the space bar, participant could move on to the following phase.

### Experiment 2: Identification of the bump number during navigation

This subsection describes an experiment of identifying the number of bumps while a participant navigated on the screen with two types of 2.5D haptic styluses. There were total five types of stimulus presented to the participants each of which consists 0–4 number of bumps, with four different sizes (2.5, 3.0, 3.5, and 4.0 cm).

The experimental procedure was designed by the experimental paradigm of the absolute identification experiment^23^. The participants were asked to stroke over a touch screen with a haptic stylus to feel the stimulus and answer the total number of the bumps that s/he felt. Since there were five stimuli, a 5-by-5 confusion matrix was numerically formed referring to the participant’s response. From the confusion matrix, we computed Information Transfer (IT), which is a correspondence measure between stimuli and the response of the participants. Then, maximum likelihood estimation of IT was derived by approximating the occurred frequency of the stimuli as follows:

4 $$\begin{aligned} IT_{est} = \sum _{j=1}^{K}\sum _{i=1}^{K} \frac{n_{ij}}{n} log_2 \left( \frac{n_{ij} \cdot n}{n_i \cdot n_j}\right) \end{aligned}$$

where *n*, $S_j$, $R_j$, $n_i$, $n_j$ , $n_{ij}$, and $S_{i}$ represent total number of trials, given stimulus, participant’s response, corresponding number of trial when stimulus $S_j$ is presented, corresponding number of trial when $R_j$ is presented by participant, the number of trials the participant responds $R_j$ to the given stimulus $S_i$. We used the variable $2^{IT_{est}}$ to derive the number of bumps of which participants could possibly identify without making the error. Chen et al.’s study provides a detailed reference on the identification experiment^23^.

A training session was provided in the same manner as the first experiment. A participant could see and feel the four different sizes of the bumps, which would be displayed in the main experiment. A caption of the triggered stimuli and corresponding visual cue for the virtual bumps was also visually shown on the screen. There was no limitation of time for examinations. The participant traced the blank screen laterally without visually displayed bumps and counted the total number of bumps in the main experiment. On each trial, the number of total bumps was randomly selected with an equal a a priori probability of 0.2 (5 alternatives). The participant was asked to type “0” to “4” for the given stimuli, indicating the number of bumps s/he felt.

The experimental computer recorded several parameters for each trial. The type of stimulus, a participant’s answer, and the trial time were stored in a log file. After each experimental run, the computer immediately calculated the sensitivity index $d'$ and the Information Transfer and recorded them to the log file.

## Results

**Figure 4:**
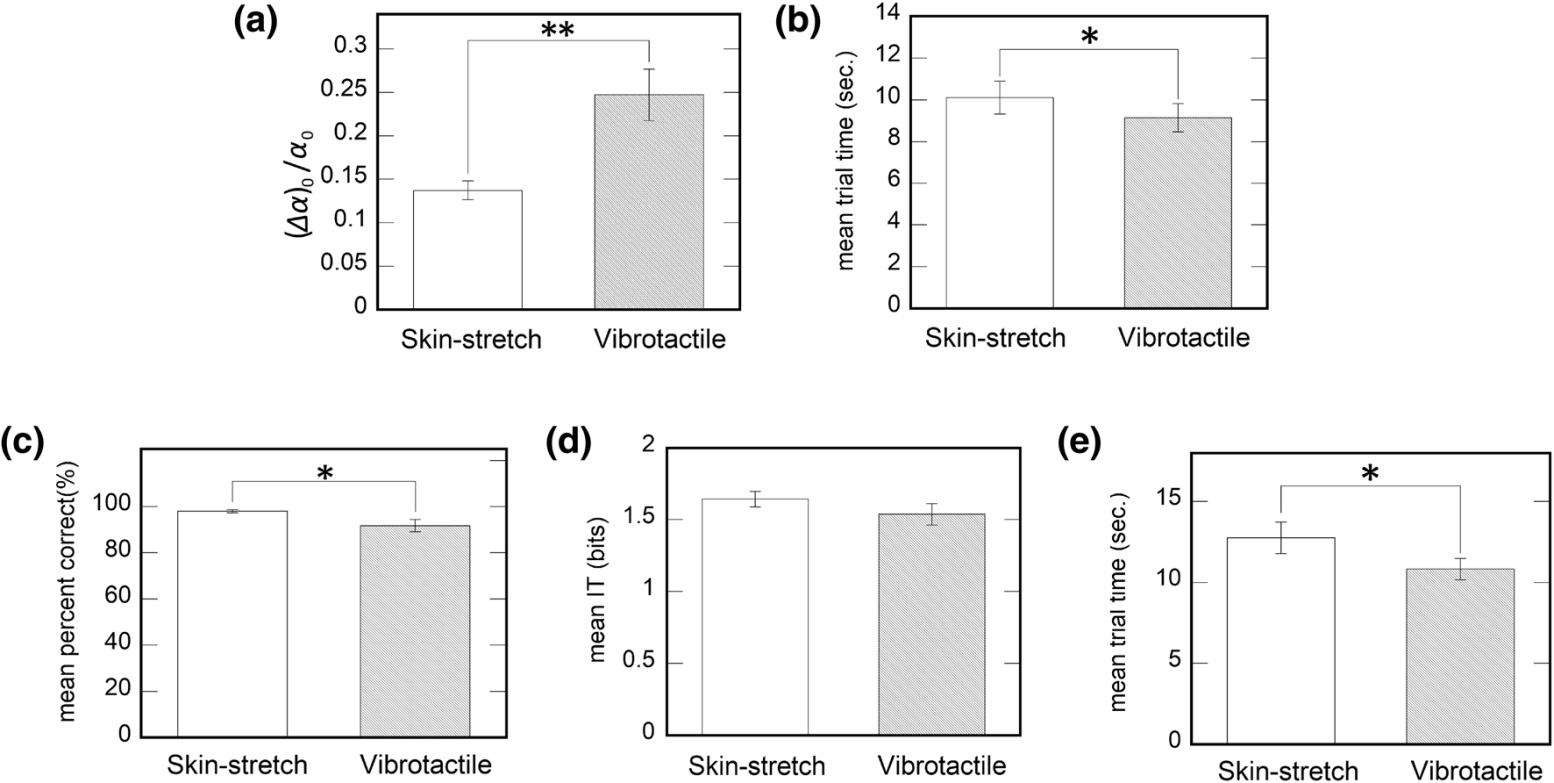
Experimental results. Error bars indicate the standard errors. (**a**,**b**) The results of Experiment 1; (**a**) The mean Weber fraction of virtual bump size perception. (**b**) The mean trial time of Experiment 1 for the two conditions. (**c**–**e**) The results of Experiment 2; (**c**) the mean percent correct score as a function of haptic stylus type. (**d**) the mean information transfer (IT) as a function of haptic stylus type. (**e**) The mean trial time of Experiment 2 for the two conditions.

We conducted two psychophysical experiments to investigate the effect of 2.5D haptic feedback on virtual object perception. The first experiment measured participants’ perception thresholds for virtual bump size by the type of 2.5D haptic feedback—lateral skin-stretch and vibration. In the second experiment, participants stroked a haptic stylus on a touch screen and counted the number of virtual bumps s/he felt, while given two different types of haptic feedback. Both experiments also evaluated the time to complete the experimental tasks by the type of 2.5D haptic feedback.

### Experiment 1: Haptic perception of 2.5D virtual bump size rendered by a haptic stylus

In the first human experiment, we evaluated the human perception of virtual bump’s size rendered on a touch screen with the two types of haptic styluses. Total 12 right-handed subjects (four females, 24–27 years old, average 25 ± 1.1 years old) were involved in the experiments. None of them had any issues with their sense of touch.

A participant stroked over a touch screen with a 2.5D haptic stylus in the main experiment and reported whether s/he felt a reference or comparison virtual bump. The experimental stimuli were virtual bumps formed as NURBS curves, rendered by either SMA-driven skin-stretch or vibrotactile stylus. We set the reference bump size to be 2.5 cm and the comparison bump size to be 3.0 cm and 3.5 cm ($\Delta \alpha$ = 0.5 cm and 1.0 cm). Hence, 4 sets of experimental runs (two haptic stylus $\times$ two $\Delta \alpha$) were conducted each of which consisted of 20 trials. The experimental computer recorded the participant’s answer and the time s/he took to complete the experimental task for each trial.

Figure 4a shows the mean Weber fraction of virtual bump size perception in Experiment 1. When we conducted a paired t-test, there was a significant difference in the bump size detection between the two types of the interface [t(11) $= -$ 3.124, *p* = 0.009]. The result suggests that the participants’ perception of bump size was more sensitive when they utilized the SMA-driven skin-stretch stylus than the vibrotactile stylus. In Fig. 4b is shown the mean trial time for the two experimental conditions. The result of a paired t-test indicates that the mean trial time was significantly longer when they used the skin-stretch stylus than the vibrotactile stylus [t(11) $=-$ 1.849, *p* = 0.046].

### Experiment 2: Identification of the bump number during navigation

In the second experiment, we tested the participants’ ability to count randomly presented virtual haptic bumps under the two experimental conditions using the styluses. The same participants as Experiment 2 took part in the experiment.

On each trial of the main experiment, the experimental computer randomly selected from 0 to 4 as the number of bumps. The size of each bump was randomly selected among four different sizes (2.5, 3.0, 3.5, and 4.0 cm). The experiment was conducted for two runs, each of which consisted of 20 trials by the type of haptic styluses. The order of the experimental run was randomized for each participant.

Figure 4c–e shows the results of Experiment 2. The result of a paired t-test indicates that the percent correct score for the SMA-driven skin-stretch stylus is significantly higher than that of the vibrotactile stylus (Fig. 4c, t(11) = 3.045, *p* = 0.011). In the meanwhile, there was no significant difference in IT between the two conditions (Fig. 4d, t(11) = 1.815, *p* = 0.097) Thus, Participants were able to perceive presence of the bumps and their numbers better when using the lateral skin-stretch stylus. Figure 4e shows the mean trial time for the two experimental conditions. As for the Experiment 1, the trial time was significantly longer for the skin-stretch stylus [t(11) $=-$ 1.918, *p* = 0.041].

### Subjective rating of the 2.5D haptic styluses

When all the experiments were over, the participants rated each method by responding to the questions regarding (1) the realism of the force (2) representation of the edge. We adopted 5-pt Likert scale for the questions each of which were balanced with negative questions. The questions of the subjective ratings were given as followings: (1) is the force from the screen realistic? (2) Is the force from the screen unrealistic? (negative question of Q1) (3) Can you perceive the start edge and the end edge of the bumps? (4) Can you not perceive the start edge and the end edge of the bumps? (negative question of Q3). We conducted paired t tests for each questionnaires between the two experimental conditions [Q1: [t(11) = 9.1, *p* < 0.001], Q2: [t(11) = 8.074, *p*< 0.001], Q3: [t(11) = 6.051, *p* < 0.001], Q4: [t(11) = 5.923, *p* < 0.001]]. Overall, the result of Q1 and Q2 implies that SMA type haptic stylus benefited the realism of contact force rendering.

## Discussion

The experimental results show that the participants could perceive the virtual bump geometry better when using a 2.5D lateral skin-stretch stylus than a vibrotactile stylus in terms of feature identification and discrimination. In Experiment 1, we tested the human perception of a virtual bump size using two haptic styluses. The results indicate that the participants could discriminate the virtual bumps size better when they were rendered with the SMA-driven skin-stretch stylus. Experiment 2 evaluated the participants’ ability to count the number of virtual bumps felt via the two types of virtual stylus. The perception score for the SMA-driven skin-stretch stylus was significantly higher than that of the vibrotactile stylus. The participants took more time to complete the experimental tasks when using the SMA-driven skin-stretch stylus than the vibrotactile stylus for both experiments. Overall, we proposed a successfully working lateral skin-stretch stylus for 2.5D haptic rendering in this paper. The experimental results indicate that participants could perceive 2.5D geometric features better in bump counting and size discrimination. Also, the participants took less experimental time with the SMA stylus than with the vibrotactile stylus.

A common trend of the two experiments’ results is that the participants could perceive the virtual bumps better with the lateral skin-stretch feedback. A possible explanation for higher sensitivity when using the lateral-skin stretch feedback can be found from the difference in the sensitivity between the two types of stimuli. The Weber fractions for the tangential force and the vibration intensity are 16%^24,25^ and 28–35%^26,27^, which implies higher sensitivity to the lateral skin-stretch than the vibratory feedback. Then, the participants of Experiment 1 would have felt the tactile feedback more salient when using the lateral skin-stretch stylus than the vibrotactile stylus. The result of Experiment 2 can be partly explained by the difference in the propagation of the stimuli. For a skin-stretch stylus, a stimulus for a contact plate is decoupled from another. For the vibrotactile stimulus, vibration from a contact plate can propagate to another via the pen body or finger skin, confusing the user about the vibration source^28^. Then, the participants of Experiment 2 would have been perplexed about the upward and downward hills of a bump.

The mean trial time for the lateral skin-stretch feedback was longer than the vibrotactile feedback for both experiments. We can find a possible explanation for the result from previous studies investigating the relationship between haptic exploration time and task accuracy. Davidson et al. studied the effect of exploration time on the haptic and visual matching of complex shapes, and they found the increased exploration time resulted in improved accuracy^29^. Similarly, the result of a haptic object size perception study revealed that the participants took longer trial time with more tactile information^30^. Thus, the previous studies hint that a user of a haptic system can take more time in a given task when more information is available, as in the case of the present study.

Another notable feature of the experimental results is high task performance for both styluses in Experiment 2. The participants recorded more than 80% of percent correct score in the virtual bump counting task. We can find an explanation for the result from the learning transfer between the physical and virtual environments^31^. Previous studies have shown that the learning transfer can occur if there is a similarity and domain knowledge between different domains^32,33^. Especially, Adams et al. found that the availability of haptic feedback can improve the learning transfer for an assembly task from the virtual to the physical environment^34^. Considering the previous studies, the participants could have benefited from earlier daily life experience in counting objects with a stylus. The haptic feedback could have helped their task during the process, because they could create similar effect of stroking over the bumps in the physical environment.

The proposed method and the present study’s findings can be applied to the HCI (Human–Computer Interaction) and haptic interface design. The experimental result indicates that the proposed lateral skin-stretch stylus can enhance the realism of the stylus stroke over a virtual feature such as an icon. Also, the stylus has the advantages such as low power consumption and compact size, which hint at the potential use for mobile devices and desktop applications. Moreover, the proposed multi-DOF haptic feedback stylus can be an alternative for force feedback haptic stylus for the CAD design, which is relatively heavy and expensive. Researchers are actively developing sensor technologies to estimate a user’s position and gesture in 3D space^35–37^. Thus, when combined with gesture recognition technologies, the haptic interface will enable a user to interact effectively with a 3D virtual environment, with a larger workspace and higher useability than a typical force-feedback haptic system.

The experimental results prove that the lateral skin-stretch feedback to a stylus can effectively render 2.5D geometry of virtual objects on a touch screen. Also, we observed a trend that more salient lateral skin-stretch feedback resulted in longer trial time than the vibrotactile feedback. Our experiment can be extended to investigate the effect of tactile feedback on more diverse surface features for 2.5D haptic feedback. For example, consider rendering a virtual bump with a size smaller than the size of the stylus tip. Then, the navigation time over the feature will be short so that rendering with vibrotactile feedback will be more efficient than the lateral haptic feedback. Convexity of virtual object shape will be another factor that can affect human haptic perception. Also, varying the combination of multi-modal haptic feedback will have a different effect on the perception of virtual features. Therefore, we are planning to generalize the present study’s findings to investigate the effect of haptic feedback on the perception of haptic features rendered on a screen. Our future work will analyze the human perception of 2.5D haptic objects by the haptic feedback type by the size and the shape of the haptic features.

## Supplementary Information

Supplementary Information.
