# Author Correction: Effect of 2.5D haptic feedback on virtual object perception via a stylus

**DOI:** 10.1038/s41598-021-00848-9

**Published:** 2021-10-27

**Authors:** Gyuwon Kim, Donghyun Hwang, Jaeyoung Park

**Affiliations:** 1 Korea Institute of Science and Technology (KIST), Center for Intelligent and Interactive Robotics, Seoul, 02792 Korea; 2 Department of Mechanical Engineering, Korea University, Seoul, 02841 Korea; 3 Department of Computer Engineering, Hongik University, Seoul, 04066 Korea

Correction to: *Scientific Reports* https://doi.org/10.1038/s41598-021-98589-2, published online 23 September 2021.

The original version of this Article contained an error in the Acknowledgments section.

“This work was supported by the National Research Foundation of Korea (NRF) Grant funded by the Korea Government (MEST) (No. NRF-2020R1A2C2014422).”

now reads:

“This work was supported by the National Research Foundation of Korea (NRF) Grant funded by the Korea Government (MSIT) (No. NRF-2020R1A2C2014422).”

The original Article has been corrected.

