# Supplementary material for: Effect of 2.5D haptic feedback on virtual object perception via a stylus
Source: Sci Rep. 2021 Sep 23;11:18954. doi: 10.1038/s41598-021-98589-2 (PMC8460700; doi:10.1038/s41598-021-98589-2)
Supplement: Supplementary file 1 — Supplementary Information. [file 41598_2021_98589_MOESM1_ESM.pdf]

SUPPLEMENTARY INFORMATION

Effect of 2.5D Haptic Feedback on Virtual Object Perception via a Stylus

Gyuwon Kim [1, 2], Donghyun Hwang [1, +], and Jaeyoung Park [3, *]

[1] Korea Institute of Science and Technology (KIST), Center for Intelligent and Interactive Robotics, Seoul, 02792, Korea

[2] Korea University, Department of Mechanical Engineering, Seoul, 02841, Korea

[3] Hongik University, Department of Computer Engineering, Seoul, 04066, Korea

* Corresponding author:

Jaeyoung Park

jypdeca@hongik.ac.kr

**Structural Specifications**		
Overall size	25 * 25 *150 mm	
Total weight	53.5 g	
DOF of the contact plate	3 – DOF (1 –DOF to each finger)	
Type and number of the actuators	3 SMA wires	
Type of the embedded sensor	Linear Potentiometer	
**Working Performance at 14V**		
Max. Displacement	*No load state*	3.132 mm
	Loaded state	2.246 mm
Force Capacity	*No load state*	3.442 N
	Loaded state	2.066 N
Heating Reaction Velocity	*No load state*	16.667 mm/s
	Loaded state	9.093 mm/s

Supplementary Table 1: The SMA Haptic Stylus Specification. Overall specifications and working performance of the SMA stylus. Total weight (53.5 g) compared to that of the conventional hand-held multi – DOF styluses are considerably small, from the range of 38 % to 71 %[1, 2]. The pen provides 3 – DOF linear motion to each finger holding the pen using 3 SMA wires and linear potentiometers. The performance of the device of maximum displacement, force capacity and reaction velocity under both no load state and loaded state shows sufficient ability delivering continuous stimuli.

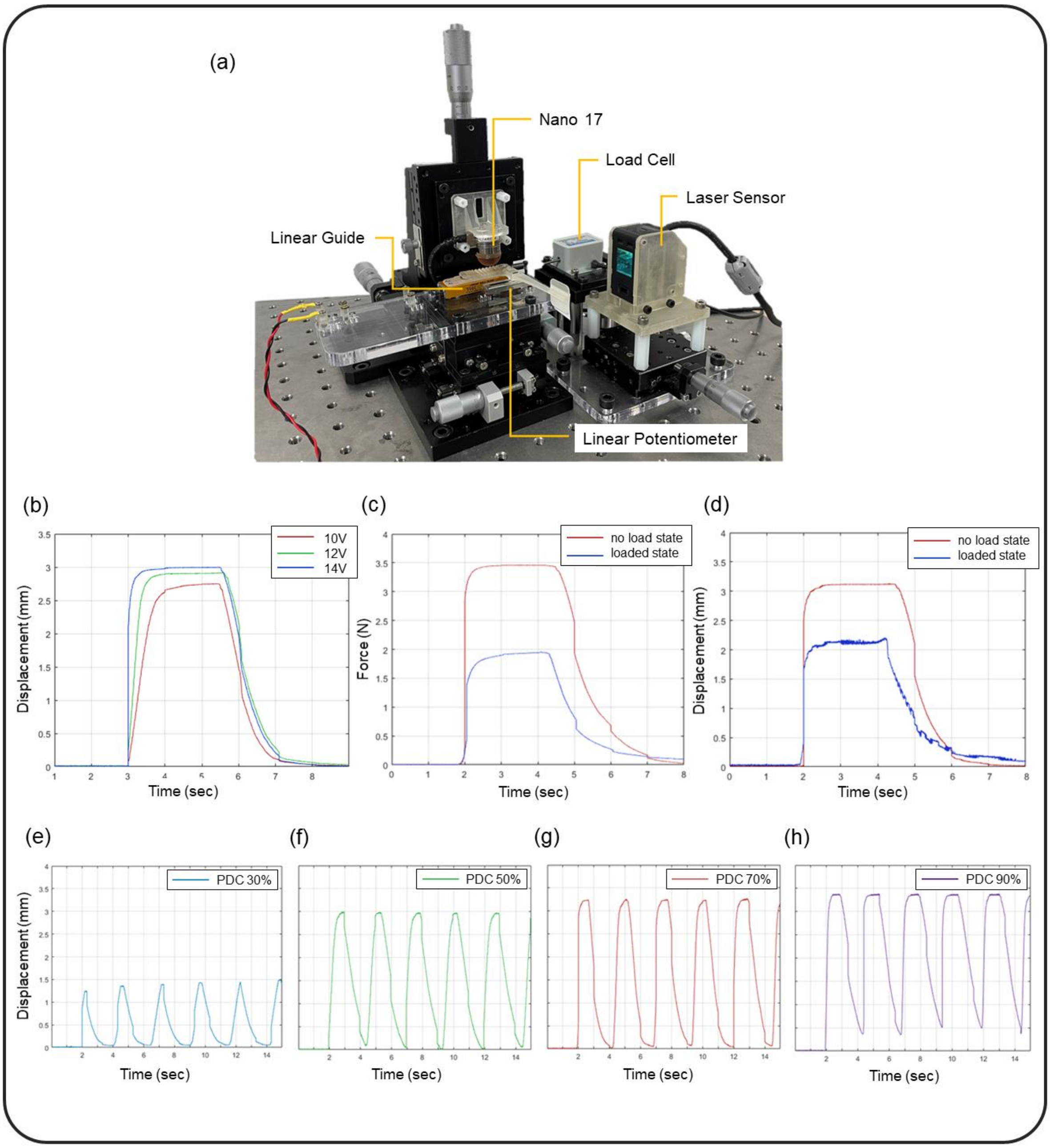

Supplementary Figure 1: The SMA Characteristics Measurement. (a) The SMA measurement environment, where laser sensor measures the displacement of the contact plate, load cell and Nano 17 each measure the SMA pulling force and the normal force applied perpendicular on the contact plate. The configuration of the SMA is identical to that of the pen. (b) A displacement result of the contact plate with applying different voltage. The higher the voltage is, the faster the plate reacts with more displacement and opposite results are shown at lower voltages. (c) - (d) A force and displacement measurement under no load state and 3 N loaded state at 14V. Under both conditions, the SMA is heated with the voltage, hold 3 seconds and is released. The results show sufficient displacement range and force to stimulate human skin under loaded state[3] exceeding the absolute threshold. (e) - (h) At the same voltage and frequency, we adopted different pulse duty cycles (PDCs) on our system and observed displacement. The proper PDC should have large displacement without error accumulations for accurate controlling. (e) and (f) shows relatively small displacement range, while (h) shows big displacement range with error accumulations. Hence, the PDC in (g) is adopted in our system.

REFREENCES

1. L. Tian, A. Song, and D. Chen, "Image-based haptic display via a novel pen-shaped haptic device on touch screens," *Multimed Tools Appl.*, 76, 14969–14992, 2017.

2. S. Kamuro, K. Minamizawa, N. Kawakami, and S. Tachi, "Ungrounded kinesthetic pen for haptic interaction with virtual environments," *IEEE International Symposium on Robot and Human Interactive Communication*, pp. 436-441, 2009.

3. D. Prattichizzo, F. Chinello, C. Pacchierotti and M. Malvezzi, "Towards Wearability in Fingertip Haptics: A 3-DoF Wearable Device for Cutaneous Force Feedback," *IEEE Transactions on Haptics*, vol. 6, no. 4, pp. 506-516, 2013.
